# Blood-brain barrier repair: potential and challenges of stem cells and exosomes in stroke treatment

**DOI:** 10.3389/fncel.2025.1536028

**Published:** 2025-04-07

**Authors:** Xiaochen Fu, Jia Li, Shoujun Yang, Jiapeng Jing, Qinzhi Zheng, Ting Zhang, Zhuo Xu

**Affiliations:** ^1^Department of Rehabilitation, China-Japan Union Hospital of Jilin University, Changchun, China; ^2^Rehabilitation Therapeutics, School of Nursing, Jilin University, Changchun, China

**Keywords:** blood brain barrier, stroke, stem cell, exosomes, cell therapy

## Abstract

Stroke is characterized with high morbidity, mortality and disability all over the world, and one of its core pathologies is blood-brain barrier (BBB) dysfunction. BBB plays a crucial physiological role in protecting brain tissues and maintaining homeostasis in central nervous system (CNS). BBB dysfunction serves as a key factor in the development of cerebral edema, inflammation, and further neurological damage in stroke patients. Currently, stem cells and their derived exosomes have shown remarkable potential in repairing the damaged BBB and improving neurological function after stroke. Stem cells repair the integrity of BBB through anti-inflammatory, antioxidant, angiogenesis and regulation of intercellular signaling mechanisms, while stem cell-derived exosomes, as natural nanocarriers, further enhance the therapeutic effect by carrying active substances such as proteins, RNAs and miRNAs. This review will present the latest research advances in stem cells and their exosomes in stroke treatment, as well as the challenges of cell source, transplantation timing, dosage, and route of administration in clinical application, aiming to discuss their mechanisms of repairing BBB integrity and potential for clinical application, and proposes future research directions. Stem cells and exosomes are expected to provide new strategies for early diagnosis and precise treatment of stroke, and promote breakthroughs in the field of stroke.

## 1 Introduction

Stroke refers to an acute disease in which blood flowing to the brain is disrupted due to sudden rupture or blockage of cerebral blood vessels, which can induce death of brain cells and damage to brain tissue. It can be categorized into ischemic and hemorrhagic stroke (Campbell and Khatri, 2020). As reported by the World Health Organization, stroke is recognized as the third leading cause of mortality on a global scale and is among the most significant contributors to prolonged disability. As indicated by data from 2024, approximately 795,000 patients have strokes annually, including around 610,000 with initial attacks and 185,000 with recurrent strokes (Martin et al., 2024). Stroke is more common in aged people, with the greatest incidence observed in individuals aged 65 and above. As the global population continues to age, stroke is expected to show a rising incidence, posing considerable strain on public health and healthcare systems.

Blood-brain barrier (BBB) serves as a permeability barrier composed mainly of brain microvascular endothelial cells, astrocytes, pericytes, and basement membranes. Primarily, it safeguards the brain from detrimental substances while maintaining the stability of the intracerebral environment. Upon an occurrence of a stroke, the integrity of the BBB is compromised, resulting in augmented extracellular fluid exudate and an intensified inflammatory response, which further exacerbates the neurological damage (Ghori et al., 2022). Disruption of the BBB has been demonstrated to be closely associated with the prognosis of stroke (Ng et al., 2022), and restoring the function of BBB is deemed as a pivotal strategy for improving the prognosis of stroke patients.

Stem cells, with advanced self-renewal capacity and multidirectional differentiation potential, have been extensively utilized in regenerative medicine and tissue repair recently. Exosomes are small extracellular vesicles, with diameters of about 30–150 nm, and are secreted by stem cells. They are capable of carrying various bioactive molecules, including proteins, lipids, and nucleic acids, and participate in intercellular signaling and material exchange (Rashed et al., 2017). Stem cell-derived exosomes exhibit a potential application in playing neuroprotective, anti-inflammatory, and regenerative effects, particularly in the context of stroke treatment. In this regard, stem cell-derived exosomes may promote the repair of neurological injury by modulating the function of BBB (Jian et al., 2019).

Despite stem cell transplantation has demonstrated a certain degree of safety, its efficacy is still not fully validated. Stem cell therapy faces several challenges, such as choice of cell source, timing of transplantation, dosage, and route of administration, all of which can impact final therapeutic outcomes (Park et al., 2020; Anthony et al., 2022; Wang Y. et al., 2024). As a developing therapeutic strategy, the mechanisms through which stem cells and exosomes function are not entirely understood, and there are ongoing issues related to standardizing their production and targeted delivery methods (Li et al., 2021; Jiang et al., 2022). In addition, the lack of sufficiently large-scale randomized controlled trials during the clinical testing phase raises concerns about the real-world effectiveness of these therapies (Chen et al., 2024). While stem cells and their derived exosomes show potential for treating stroke, they still face various challenges, including uncertainties about their efficacy, complexities in the production process, and standardization issues in clinical applications. Therefore, this article intends to provide a review of literature on stroke associated BBB studies and to examine the potential therapeutic role of stem cells and their exosomes on stroke, and then summarize the current research progress and propose the future research directions.

## 2 Overview of stroke

### 2.1 Stroke classification and mechanisms

#### 2.1.1 Ischemic stroke

Ischemic stroke represents the most prevalent form of stroke, constituting 62.4% of all new stroke cases globally in 2019, as reported by the 2019 GBD study (Martin et al., 2024). The etiology of ischemic stroke is predominantly attributed to a decrease or cessation of cerebral blood flow, often precipitated by the narrowing or occlusion of blood vessels resulting from thrombosis or atherosclerosis. The TOAST classification system further categorizes ischemic stroke into various sub-types, including large-artery atherosclerosis, cardiac embolism, small-vessel occlusion, acute stroke of other determined etiology, and stroke of undetermined etiology (Adams et al., 1993).

In the context of ischemic stroke, the onset of hypoxia precipitates a disruption in cellular metabolism, culminating in cellular death and subsequent brain tissue damage. The pathogenesis of ischemic stroke is associated with a variety of factors, including vascular endothelial dysfunction, inflammatory response, and changes in blood rheology. Within 24 h of the onset of ischemia, brain tissues exhibit a substantial inflammatory response, accompanied by a significant escalation in pro-inflammatory cytokine levels, such as IL-1β and TNF-α, which further exacerbate brain injury (Ramiro et al., 2018). Ischemic stroke also triggers oxidative stress, which leads to the production of free radicals that damage cell membranes and DNA, thereby exacerbating cell death (Allen and Bayraktutan, 2009). Furthermore, research has identified a correlation between blood biomarkers, such as D-dimer and C-reactive protein, in patients with ischemic strokes and the underlying mechanisms of stroke. These biomarkers have been shown to be indicative of the pathophysiological status of different ischemic strokes (Katan and Elkind, 2018).

#### 2.1.2 Hemorrhagic stroke

The classification of hemorrhagic stroke principally encompasses two distinct categories: intracerebral hemorrhage (ICH) and subarachnoid hemorrhage (SAH). According to the 2019 GBD study, ICH accounts for 27.9%, and SAH accounts for 9.7% (Martin et al., 2024). ICH is defined as the accumulation of blood in the brain tissue, with underlying causes including high blood pressure, ruptured cerebral aneurysms, and blood disorders. SAH, characterized by the entry of blood into the subarachnoid space of the meninges, is typically caused by a ruptured aneurysm. Research has demonstrated that patients afflicted with hemorrhagic stroke frequently exhibit a suboptimal prognosis and an elevated risk of recurrence. Consequently, the timely identification and management of risk factors are of paramount importance (Banerjee et al., 2020; Williams et al., 2023).

With regard to the underlying mechanisms, the occurrence of ICH is typically accompanied by the rupture of blood vessels and the leakage of blood, resulting in direct and secondary damage to brain tissue. Following the hemorrhage, the formation of a hematoma results in an increase in local pressure. The hemoglobin breakdown products within the hematoma can trigger secondary injury, leading to ischemia and cerebral edema in the surrounding brain tissue (Chen et al., 2021). Concurrent studies have demonstrated a close correlation between the activation of inflammatory responses and neuronal death, with the subsequent release of inflammatory mediators exacerbating brain injury (Dong et al., 2024).

### 2.2 Influence of gender and age on the incidence of stroke

Significant disparities in the incidence and clinical manifestation of stroke have been observed between sexes. Research has demonstrated that the overall incidence of stroke is consistently higher in males compared to females, particularly within younger and middle-aged demographics (Thomas et al., 2021). However, hormonal changes after menopause significantly increase the risk of stroke in women, and common risk factors such as hypertension, atrial fibrillation, and diabetes mellitus are more prevalent in women (Wassertheil-Smoller et al., 2003). Women have been shown to have a more unfavorable stroke prognosis, as indicated by higher mortality and functional impairment rates (Branyan and Sohrabji, 2020). This may be attributable to a number of factors, including their advanced age at the time of onset, the presence of more concomitant illnesses, and the tendency to present with more atypical symptoms compared to men, which can result in delays in diagnosis and treatment. Consequently, there is an imperative to comprehend gender-related disparities to facilitate the development of customized prevention and treatment strategies for diverse patient populations.

The incidence of stroke also varies significantly across age groups. Research has demonstrated a positive correlation between age and stroke incidence, with a notable surge observed among individuals aged 65 and above. According to the 2019 data, the age distribution of stroke shows a significant peak, concentrated in the 70+ age group, where the incidence is generally higher in men than in women (GBD 2019 Stroke Collaborators, 2021). Although stroke incidence is lower in younger individuals (under 50 years of age), recent studies have documented an increase in stroke events in this demographic due to lifestyle changes and an elevated risk profile (Ekker et al., 2018). This distribution pattern underscores the necessity for the development of suitable prevention and intervention strategies that are tailored to address the shifting stroke incidence across diverse age groups.

### 2.3 Current status of preclinical modeling applications for stroke

#### 2.3.1 The use of animal models in ischemic stroke research

**FIGURE 1:**
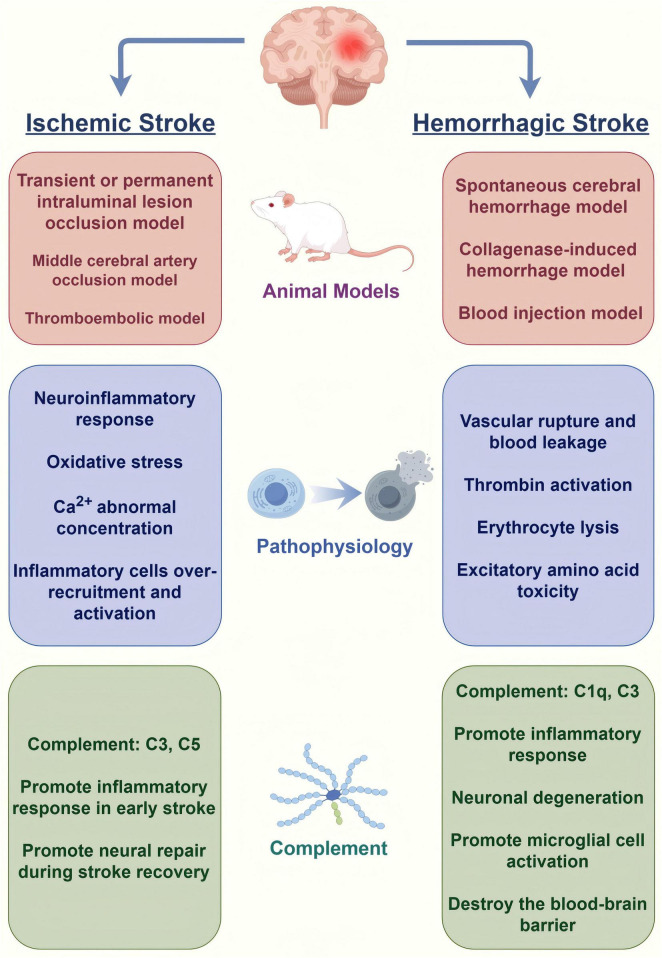
Models, pathophysiology and the role of complement system of stroke (by Figdraw).

Animal models play a critical role in ischemic stroke research because of their ability to mimic the physiological and pathological features of human stroke (Figure 1). Transient or permanent intraluminal lesion occlusion, middle cerebral artery occlusion (MCAO), and thromboembolic models are the most commonly used models to simulate human ischemic stroke. These models can effectively reproduce the major physiological changes and pathological processes of ischemic stroke. MCAO models are the main models used to study ischemic stroke, and studies have shown that they can be used not only to evaluate the efficacy of new therapies, but also to study the mechanisms of neuroprotection after stroke (Li and Zhang, 2021). The reproducibility and controllability of the MCAO model make it an important tool for drug screening and mechanistic studies. The reproducibility and controllability of the MCAO model make it an important tool for drug screening and mechanistic studies. In recent years, researchers have optimized the model, such as using microcatheter technology for local embolization, to improve the accuracy and reproducibility of the model (Komatsu et al., 2021). In addition to the commonly used rodents, larger animal models such as pigs and non-human primates have been used in ischemic stroke research, which are considered more clinically relevant due to the similarity of their brain structure to humans and are better able to mimic the pathophysiology of stroke in humans (Taha et al., 2022). Using these animal models, researchers can explore new therapeutic strategies for ischemic stroke and evaluate the safety and efficacy of drugs, thereby advancing translational clinical research.

#### 2.3.2 Animal models in the study of hemorrhagic stroke

Hemorrhagic stroke research also relies on animal models, particularly to study the pathological mechanisms and therapeutic strategies of hemorrhagic stroke. Common animal models include spontaneous cerebral hemorrhage models, collagenase-induced hemorrhage models, and blood injection models that mimic the pathology of human hemorrhagic stroke (Chen et al., 2021; Li and Zhang, 2021). Studies have shown that the use of these models can be effective in evaluating the potential of novel drugs to reduce post-hemorrhagic brain injury and improve functional outcomes. In addition, alterations in the microenvironment after hemorrhagic stroke have been found to be closely related to the inflammatory response, providing new ideas for the development of novel therapies (Harjunpää et al., 2024). Through in-depth study of animal models, scientists can better understand the mechanism of hemorrhagic stroke and provide a theoretical basis for clinical treatment.

#### 2.3.3 Integration of cellular models with *in vitro* experiments

The development of *in vitro* models has provided another effective tool for preclinical stroke research, and the combination of cellular models and *in vitro* experiments is playing an increasingly important role in stroke research. In recent years, advances in cell culture technology, particularly the advent of three-dimensional cell culture systems, have allowed researchers to better mimic the *in vivo* microenvironment and thus improve the prediction of drug responses. Using human induced pluripotent stem cell (iPSC)-derived organoid models, researchers can recapitulate the pathological features of neurodegenerative diseases *in vitro* to assess the efficacy and safety of new drugs. These models allow researchers to study cellular responses to ischemia and hemorrhage in a controlled environment, providing insight into the mechanisms of stroke (Pang et al., 2024). *In vitro* culture models of human neurons or glial cells can be used to simulate physiological changes in cells after ischemia or hemorrhage and to assess the effects of drugs on cell survival and function. In addition, the combination of using co-culture models of multiple cell types can better simulate the microenvironment in the brain and help to study cell-cell interactions and their role in stroke (Song et al., 2021). This combination of cellular modeling and *in vitro* experiments not only improves the reproducibility and reliability of studies, but also provides an important experimental basis for the development of new therapies and advances in stroke research (Van Breedam and Ponsaerts, 2022). Despite the advantages of *in vitro* models, they are still not a complete replacement for animal models because the *in vitro* environment cannot fully mimic the complex cellular interactions and signaling that occur under physiological conditions (Peng et al., 2021).

### 2.4 Role of the complement system in stroke

#### 2.4.1 Complement activation in stroke

After ischemic stroke occurs, activation of the complement system can lead to a series of inflammatory responses that further exacerbate brain tissue damage. Excessive activation of complement leads to disruption of the blood-brain barrier, further exacerbating neuronal damage and death. The levels of complement components such as C3 and C5 are significantly elevated after ischemia, which enhances the local inflammatory response by promoting the infiltration and activation of inflammatory cells (Ma et al., 2019). C3a and C5a, as the key effectors of complement activation, are able to induce the activation of microglia and promote the release of inflammatory factors, which aggravate the brain tissue injury and attract more immune cells to reach the injury site (Ahmad et al., 2019; Couch et al., 2023).

After the onset of hemorrhagic stroke, the activation of complement can exacerbate the inflammatory response and cerebrovascular permeability, which can lead to the exacerbation of secondary brain damage. Elevated complement component C4 is strongly correlated with the severity of neurological deficits, suggesting that complement may serve as a biomarker for prognostic assessment in hemorrhagic stroke (Wu et al., 2023). Complement activation has also been associated with the formation of perihematomal edema, which is considered to be one of the important factors in the poor prognosis after hemorrhagic stroke (Hatchell et al., 2023).

#### 2.4.2 Effect of complement on nerve damage

The complement system plays an important role in nerve injury by regulating apoptosis, affecting blood-brain barrier integrity, and promoting axonal remodeling. Complement activation leads to an increased inflammatory response in neuronal cells, releasing a variety of proinflammatory factors that bind to specific receptors, promoting microglia activation, increasing neuroinflammatory responses, and ultimately leading to neuronal apoptosis (Kakarla et al., 2021). Elevated levels of complement cleavage products, such as C3a and C5a, are directly proportional to the degree of disruption of the blood-brain barrier, and inhibition of complement can significantly reduce the extent of blood-brain barrier damage and inflammatory response, and improved neurological function (Holste et al., 2021). Complement components C1q and C3 play a facilitating role in axonal remodeling and influence neural repair and regeneration (Kanmogne and Klein, 2021). Thus, regulation of the complement system may provide new strategies for the treatment of nerve injury.

#### 2.4.3 Role of complement in the post-stroke inflammatory response

There is a complex interaction between the complement system and inflammatory factors. Complement component C1 inhibitors and other anti-inflammatory factors can inhibit excessive inflammatory responses after ischemia and promote tissue repair and regeneration (Ma et al., 2019). Activation of complement components C3 and C5 can stimulate macrophages and other immune cells to release pro-inflammatory cytokines, which in turn promote further complement activation. While the production of C3a and C5a promotes the infiltration of inflammatory cells, they can also play an anti-inflammatory role by modulating the polarization state of macrophages and promoting the production of M2-type macrophages (Alawieh et al., 2020). This interaction is not only significant in the acute phase, but may also play an important role in the recovery process after stroke, affecting the ability of nerve repair and regeneration.

### 2.5 Current treatment strategies for stroke

Existing treatment strategies for hemorrhagic stroke, current treatment strategies mainly include measures such as surgery to control bleeding and reduce intracranial pressure. The complexity of the surgery and the overall health status of the patient also affects the outcome of postoperative recovery, and some patients may face a higher risk of mortality and complications after surgery (Al-Kawaz et al., 2020). In ischemic stroke the main reliance is on thrombolytic therapy and mechanical retrieval of thrombus. Recombinant tissue plasminogen activator (rt-PA), is now widely recognized as a thrombolytic therapy treatment. Thrombolysis works best usually within 3–4.5 h of symptom onset (Wang et al., 2019). Prompt thrombolysis significantly reduces patient disability and mortality. Beyond this time window, treatment is significantly less effective and may even lead to greater risk (Bangad et al., 2023; Jazayeri et al., 2024). Mechanical thrombectomy provides a better treatment option for patients with large vessel occlusions, but its effectiveness also decreases over time (Albers et al., 2021; Yeo et al., 2021). In addition, anticoagulants in the available pharmacological treatments may have bleeding complications in some patients, especially in high-risk patients, while others may fail to achieve the desired anticoagulant effect (Huang et al., 2015; Paciaroni et al., 2015).

These traditional therapies often only address the symptoms and fail to repair the damaged nerve tissue at the root of the problem, and may be accompanied by certain side effects, which makes it especially important to find new treatments. Stem cell therapy has achieved favorable results in preclinical and early clinical studies by promoting nerve regeneration, inhibiting inflammatory response and improving blood supply, among other mechanisms. Therefore, incorporating stem cell and exosome therapies into the comprehensive treatment program of stroke can bring new hope and better therapeutic results for patients.

### 2.6 Dysregulated signaling pathways after stroke

Although the pathophysiological mechanisms of hemorrhagic and ischemic stroke are different, both emphasize the importance of dysregulated cellular signaling pathways in stroke pathogenesis. In the study of these signaling pathways, it has been found that various factors including inflammatory response, oxidative stress, apoptosis, neuronal regeneration and excitotoxicity are closely related to post-stroke dysfunction and BBB damage. These signaling pathways are not only intertwined, but may also exhibit different functions at different time points after stroke. Specific details are shown in the table below (Table 1). How effectively these signaling pathways are regulated in future research and clinical interventions will be key to improving prognosis after stroke.

**TABLE 1:** Key signaling pathways dysregulated in stroke.

Pathological reaction	Signaling pathway	Exception mechanism	Effect	References
Inflammatory Response	NF-κB	IKK complex ↑ leading to IκB ↓ NF-κB enters the nucleus	Promoting the expression of IL-6, TNF-α and COX-2	Lu et al., 2024
	NLRP3	ROS and ATP activate NLRP3 Caspase-1 activation ↑ Leading to IL-1β and IL-18 ↓	Triggers inflammatory response and cellular pyroptosis	Fann et al., 2013
	TLR4/MyD88	Activated Toll-like receptor 4 (TLR4) by damage-associated molecular patterns (DAMPs) Activate NF-κB via MyD88-dependent pathway	Aggravate neuroinflammation	Balch et al., 2020; Hao et al., 2024; Zhang et al., 2024
Oxidative stress	Nrf2/ARE	Nrf2 dissociates from Keap1 and enters the nucleus to activate the antioxidant response element (ARE)	Promote the expression of HO-1 and SOD	Zhang et al., 2019; Liu et al., 2024
	NOX	Activates NADPH oxidase (NOX), generating large amounts of ROS	Oxidative damage to lipids, proteins and DNA	Kahles and Brandes, 2013
Cell death	Notch	Release of NICD Promote p53 stability and signaling	Neuronal apoptosis	Balaganapathy et al., 2018
	TNF-α	TNF-α binds to death receptors and activates caspase-8, which in turn activates caspase-3	Triggers apoptosis	Velier et al., 1999; Datta et al., 2020
	mTOR	mTOR pathway ↓ express autophagy-related proteins ↑	Excessive autophagy leads to cell death	Perez-Alvarez et al., 2018
	NLRP3 inflammasome	Activates Caspase-1, cleaves GSDMD	Induces cellular pyroptosis	Wang et al., 2021; Long et al., 2023; Yang H. et al., 2023
Neuroprotection	PI3K/Akt	Neurotrophic factors (e.g., BDNF) activate PI3K, which in turn activates Akt	Inhibits apoptosis and promotes cell survival	Hu et al., 2022
	MAPK/ERK1/2	Growth factor activates the ERK cascade reaction	Promotes neuronal survival and synaptic plasticity	Narasimhan et al., 2009
	HIF-1	Ischemia and hypoxia induce stable expression of HIF-1	Promote angiogenesis and energy metabolism adaptation	Semenza, 2009
Excitotoxicity	DAPK1	NMDAR overactivation promotes Ca^2+^ influx	Activate calcium-dependent enzymes	Qin et al., 2022
	CP-AMPAR receptor	Ca^2+^ overload Zn^2+^ accumulate in neurons	Aggravate neuronal damage	Lu et al., 2023
Nerve repair and regeneration	Wnt/β-catenin	Wnt ligand binds to the receptor Inhibits β-catenin degradation	Promote neural stem cell proliferation and differentiation	Yadava et al., 2025
	Notch	Activate target genes upon entry into the nucleus	Regulation of neural stem cell self-renewal and differentiation	Jha et al., 2022
Blood brain barrier disruption	P38/MAPK/SGK1	Increase p38/MAPK phosphorylation, activate downstream targets SGK13.	Downregulates tight junction proteins	Zhang et al., 2015
	PKC	Activate PKC-θ and PKC-δ isozymes	Reversibly disrupt occludin, claudin-5, and ZO-1	Zheng et al., 2023
	PKG	Hypoxia induces VEGF expression Activate phospholipase Cγ (PLCγ)	Increase vascular permeability Disrupt the continuous localization of ZO-1 and ZO-2 at the cell membrane	Fischer et al., 2004

## 3 Influence of stroke on BBB

### 3.1 Structure and function of BBB

Blood-brain barrier is a natural barrier between the brain and circulatory system. BBB is composed of tightly connected endothelial cells, basement membranes, pericytes, and peduncles of astrocytes, forming a complex and highly regulated system. The primary function of BBB is to modulate the flow of substances into and out of the brain, safeguard the central nervous system (CNS) from harmful substances, and permit the passage of essential nutrients and metabolites. As report, BBB is the largest interface for molecular blood-brain exchange (Nag and Begley, 2005). BBB integrity is of paramount importance for maintaining normal functions of nervous system. On the other hand, damaged structure and impaired function of BBB can result in neuronal damage and neurological dysfunction.

Tight junctions in brain microvascular endothelial cells (BMECs) are protein complexes that facilitate the adhesion of neighboring barrier cells. Based on subcellular localization, tight junctions can be categorized into transmembrane proteins and cytoplasmic proteins. Tight junction transmembrane proteins between neighboring cells bind to each other through extracellular loops, forming a “zipper” structure that closes the paracellular gap. Cytoplasmic proteins, on the other hand, act as bridges connecting transmembrane proteins and cytoskeletal proteins, forming a complete meshwork that provides structural support for maintaining BBB integrity. The BBB achieves its selective permeability through tight junctions among barrier cells, thereby influencing exchange of substances between blood and cerebrospinal fluid (Banks et al., 2021). Furthermore, absence of pericytes results in the loss of BBB integrity during embryogenesis (Daneman et al., 2010). Laminin in astrocytes regulates pericyte differentiation and maturation and induces/maintains tight junction protein expression in endothelial cells, thereby promoting BBB integrity (Yao et al., 2014). The interaction between pericytes and astrocytes stabilizes the function of BBB and greatly contributes to maintaining BBB integrity.

### 3.2 Structural and functional changes in BBB after stroke

**FIGURE 2:**
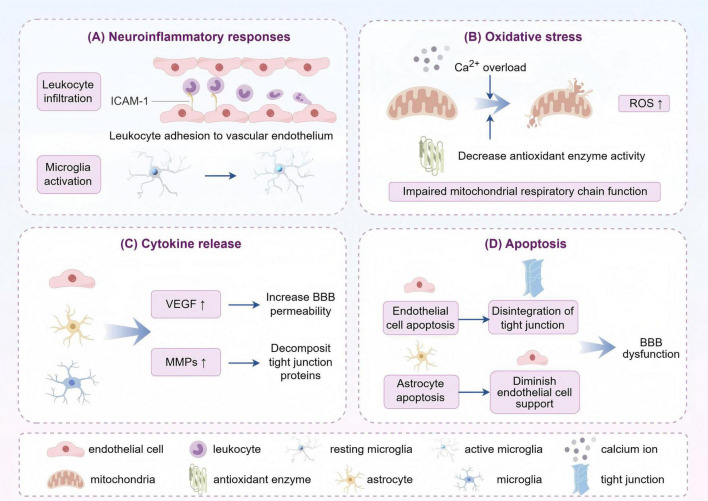
The primary mechanisms of post-stroke blood-brain barrier (BBB) impairment can be categorized as follows: **(A)** Inflammatory response: leukocyte infiltration and microglia activation after stroke release inflammatory mediators that damage the endothelial cells and tight junctions of the BBB and increase its permeability. **(B)** Oxidative stress: stroke leads to impaired energy metabolism in the brain, elevated reactive oxygen species (ROS) production, and impaired antioxidant defense system. ROS attack the components of the BBB, destroying its structure and function. **(C)** Cytokine release: after stroke, cytokines such as vascular endothelial growth factor (VEGF) and matrix metalloproteinases (MMPs) are released, the former activating relevant receptors to open tight junctions, and the latter degrading extracellular matrix and tight junction proteins to disrupt the integrity of the BBB. **(D)** Apoptosis: stroke-induced factors induce apoptosis or abnormal function of blood-brain barrier endothelial cells and astrocytes, leading to structural disintegration and destabilization of the BBB (by Figdraw).

The mechanism of BBB injury at the onset of stroke is multifactorial, involving neuroinflammatory responses, oxidative stress, cytokine release, and apoptosis (Figure 2).

In the aftermath of a stroke, structure and function of BBB change substantially, predominantly manifested as decreased expression of tight junctions and an augmentation in permeability of endothelial cells. During the acute phase of ischemia, microglia are activated to secrete cytokines such as TNF-α, IL-1β, and IL-6, which further exacerbate the destruction of BBB (Guo et al., 2023). Furthermore, neuronal hypoxia and metabolic imbalance impair the function of endothelial cells, which aggravates BBB dysfunction and promotes the formation of brain edema. For example, after acute ischemic stroke, inadequate O_2_ delivery will occur due to insufficient blood flow, which in turn leads to ATP deficiency and ion transport dysfunction, thereby causing excessive glutamate release and contributing to astrocyte swelling (Sun et al., 2019). Meanwhile, intracellular Ca^2+^ overload induces mitochondrial and endoplasmic reticulum dysfunction, allowing a rapid increase in reactive oxygen species, which swiftly inhibits antioxidant defenses and give rise to further tissue damage (Rodrigo et al., 2013; Jiang et al., 2018). BBB disruption can give profound influence on neurological function. Accordingly, safeguarding the structural integrity and function of BBB represents a crucial strategy for enhancing stroke prognosis and facilitating neurological recovery (Guo et al., 2023).

Blood-brain barrier damage after ischemic stroke is usually a gradual process, whereas hemorrhagic stroke can cause significant damage in a short period of time. After hemorrhagic stroke, local inflammation and cellular damage due to direct entry of blood components into brain tissue and hematoma formation further exacerbate BBB destruction, leading to more severe cerebral edema and neurological deficits (Shtaya et al., 2021). As the hematoma expands, the release of hemoglobin and other blood components triggers a strong local inflammatory response that further activates microglia and other immune cells, releasing large amounts of pro-inflammatory cytokines that lead to endothelial cell apoptosis and an increase in blood-brain barrier permeability, resulting in increased cerebral edema and neuronal cell death, and ultimately affecting the neurological recovery of the patient (Alsbrook et al., 2023).

## 4 Basic concepts of stem cells and exosomes

### 4.1 Definition and clinical application of stem cells

**FIGURE 3:**
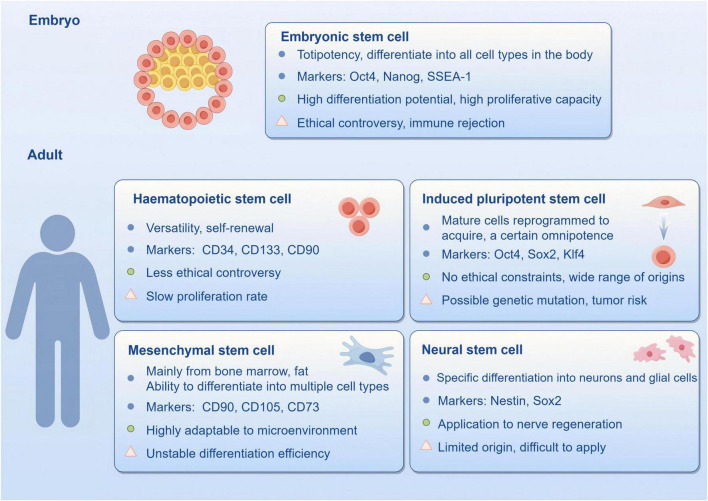
Nature markers, advantages and disadvantages of stem cells (by Figdraw).

Stem cells have achieved extensive utilization in medicine, including regenerative medicine, tissue engineering and the disease treatment which are currently attracting significant attention in modern medical science due to their promising potential. According to their origin, stem cells can be categorized into embryonic stem cells (ESC), mesenchymal stem cells (MSC), induced pluripotent stem cells (iPSC), neural stem cells (NSC), hematopoietic stem cells, and so on. Each type of stem cell differs in biological properties, application potential and clinical prospects (Figure 3). The capacity of stem cells to self-renew and differentiate in multiple directions makes them well-suited to replacing damaged or dead neurons, secreting neurotrophic factors, regulating immune responses, and promoting endogenous neurogenesis, thereby improving pathological conditions. In recent years, there have been notable successes in utilizing stem cell therapy in CNS-related diseases (Ying et al., 2023). To illustrate, MSCs have been demonstrated to effectively inhibit ferroptosis and facilitate functional recovery following spinal cord injury through mitochondrial transfer, exhibiting robust regenerative capacity and safety (Yao et al., 2023). Furthermore, research on using stem cells to treat neurodegenerative diseases and traumatic brain injury is advancing, with numerous trials yielding positive outcomes (Sivandzade and Cucullo, 2021; Zhou et al., 2024). Despite the promising outlook for stem cell therapy, it is still confronted with significant challenges, including ethical concerns, the standardization of cell sources, and the long-term safety of the therapy (Nazim and Ahmad, 2023).

### 4.2 Biological properties of exosomes and their role in cellular communication

**FIGURE 4:**
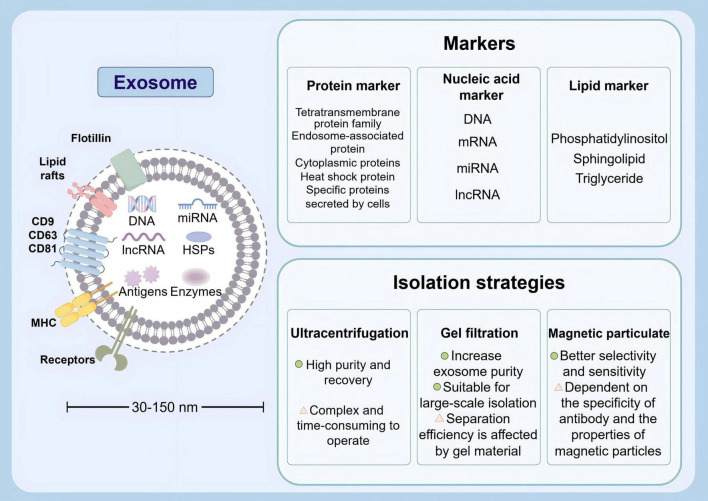
Physical characteristics, various markers and isolation strategies of exosomes (by Figdraw).

Exosomes are nanosized vesicles secreted by cells, usually between 30 and 150 nm in diameter, and are widely found in a variety of biological fluids. These tiny vesicles contain biologically active molecules such as proteins, lipids, and RNA. Their specific markers include transmembrane proteins (CD9, CD63, CD81) and heat shock proteins (HSP70). In consideration of their pivotal function in intercellular communication, exosomes can transmit information and regulate the function of target cells (Bebelman et al., 2018). Exosomes were firstly introduced in the early 1980s, with the description of small vesicles formed during the maturation of reticulocytes. These vesicles were proposed to play a role in selective externalization and removal of transferrin receptors (Pan and Johnstone, 1983; Johnstone et al., 1987). These years, exosomes are recognized to serve as carriers that facilitate intercellular communication through exchange of proteins and genetic material. The intrinsic characterizations of exosomes in regulating various intracellular pathways enhance their use in therapeutics in tumors, traumatic brain injury, and neurodegenerative diseases (Katakowski et al., 2013; Perets et al., 2019; Chen H.-X. et al., 2020; Zhong et al., 2023). Through rigorous scientific inquiry into exosomes, researchers aspire to devise innovative therapeutic strategies leveraging these naturally occurring nanoscale carriers. Strategies for exosome isolation mainly include ultracentrifugation (the most commonly used), gel filtration, magnetic bead method, etc. Each method has its own advantages and disadvantages in terms of purity, yield, and operational complexity, and the appropriate method needs to be selected according to the research needs (Figure 4). However, the complex heterogeneity of exosomes and the standardization of isolation and purification techniques still require further investigation to facilitate their clinical utilization (Li et al., 2017; Vargas-Rodríguez et al., 2023; Yang H. et al., 2023). The composition of exosomes has been demonstrated to vary depending on the state of their parent cells, with those from different sources exhibiting disparate biological effects (Théry et al., 2002). This article will focus on the role of stem cell-derived exosomes on BBB damage.

## 5 Role of stem cells on BBB after stroke

### 5.1 Stem cells in stroke treatment

Using stem cells to treat stroke is emerges as a topic of growing interest in neuroscience. A substantial body of evidence from scientific studies has demonstrated that various sources of stem cells, including mesenchymal stem cells and neural stem cells, exhibit possibility to enhance neuronal repair and facilitate neurovascular repair following a stroke (Boese et al., 2020; Do et al., 2021; Hamblin and Lee, 2021; Hamblin et al., 2021; Yoshida et al., 2021; Datta et al., 2022; Kvistad et al., 2022; Liu et al., 2023). These findings have been corroborated through *in vivo* and *in vitro* experimentation (Table 2).

**TABLE 2:** Stem cells in stroke treatment.

Participants	Cell type	Outcome assessment	Limitation	References
**Preclinical study**				
PIT-BD mice	ESCs	Enhance angiogenesis in endogenous endothelial cells	Risk of tumor development	Nagai et al., 2010
MCAO rats	ESC-NSCs	Differentiate neurons, astrocytes, oligodendrocytes and endothelial cells No chromosomal abnormalities or tumor formation	–	Daadi et al., 2008
MCAO rats	ESC-NPCs	Slight improvement in sensorimotor function	No change in infarct size Low survival of inhibitory cells	Hicks et al., 2009
MCAO mice	iPSCs-NSCs	Rapid migration to damaged areas Ameliorate BBB damage	Limited range of cell migration	Eckert et al., 2015
MCAO rats	MSCs-Exo	Reduce microglia activation Promote BBB integrity	Differences in biological functions of MSCs from different sources Gender difference	Venkat et al., 2020
MCAO mice	hAMSCs	Improve neurological deficits Reduce BBB disruption and apoptosis in the peri-infarct region	Variability of the injection target point	Yoshida et al., 2021
Collagenase-induced ICH mice	ADSCs-Exo	Attenuate hemin-induced cell injury and ferroptosis Improve neurologic function	–	Yi and Tang, 2021
Autologous whole blood injection ICH mice	MSCs	Modulate of microglia/macrophage polarization Accelerate hematoma clearance	Uncertain whether it provides a protective effect at an early stage or promotes myelin regeneration of nerve fibers at a later stage	Gao et al., 2022
Double blood injection SAH rats	hucMSCs-Exo	Regulate of neurological function, brain edema and neuronal apoptosis	–	Zhao et al., 2019
**Clinical study**				
Carotid ischemic stroke	MSCs	Safe and feasible Facilitate motor recovery through sensorimotor neuroplasticity	Delays in administration are limited by varying dosage requirements	Jaillard et al., 2020
Acute, cortical ischemic stroke	UCBs	Downregulate inflammation and promote neuroprotection and plasticity Improved functional outcome at 3 months follow-up	–	Laskowitz et al., 2018
Ischemic stroke (1 acute stage, 3 subacute stage, 2 stroke sequelae)	MSCs and NSCs	Improve neurological functions, disability levels, and daily living abilities No tumors at 2 years follow-up	Low-grade fever within 24 h	Qiao et al., 2014
Supratentorial ischemic stroke	NSCs	Improve upper limb function	Serious adverse events (non-specific immune response, seizures and sepsis)	Muir et al., 2020
Acute ICH	Autologous bone marrow mononuclear cell and MSCs	Safe and feasible Significant improvement in neurological function at 6 months	Serious adverse events (five low-grade fever and one lung cancer 4 months after the MSCs implantation)	Li et al., 2013
IVH	Human UCB-derived MSCs	Safe and feasible No mortality in MSC transplantation group	Need to determine the optimal dose, timing, and route of cell transplantation	Ahn et al., 2018

PIT-BD, photochemically induced thrombotic brain damage; ESC, embryonic stem cell; MCAO, middle cerebral artery; NSC, neural stem cell; NPC, neural progenitor cell; iPSC, induced pluripotent stem cell; BBB, blood brain barrier; MSC, mesenchymal stem cell; MSCs-Exo, exosomes derived from MSCs; hAMSC, human amniotic mesenchymal stem cell; ICH, intracerebral hemorrhage; ADSC, adipose-derived stem cell; SAH, subarachnoid hemorrhage; hucMSCs, human umbilical cord mesenchymal stem cells; UCB, umbilical cord blood; IVH, Intraventricular hemorrhage.

#### 5.1.1 Stem cell transplantation

In the context of stroke therapy, stem cells can be administrated through three routes: intravenous (IV), intra-arterial (IA), and intranasal (IN). IN route, which offers numerous advantages over the other two pathways, was firstly proposed in 2013 in research focusing on ischemic stroke (Wei et al., 2013; Table 3).

**TABLE 3:** Delivery routes of stem cell therapy in stroke.

Routes	Advantages	Limitations	References
Intravenous	Easy operation	Retention in the lungs due to pulmonary first-pass effect	Fischer et al., 2009
Intra-arterial	Minimally invasive More diffuse drug distribution patterns Higher number of cells transplanted in the target brain region	Vascular obstruction may occur in small arteries and capillaries	Walczak et al., 2008; Li et al., 2010; Yavagal et al., 2014
Intranasal	Non-invasive Circumvent the effects of different needle diameters and flow rates on stem cell viability Direct transport of drugs into the brain Patients can tolerate high-frequency administration	Unique and complex structure and function of the nasal cavity pose challenge to endogenous delivery of drugs into the brain	Tol et al., 2008; Miyake and Bleier, 2015; Chau et al., 2018; Galeano et al., 2018; Li et al., 2022

Intranasal delivery enables stem cells to traverse the olfactory epithelium and gain access to the space in close proximity to the ostium of the turbinate. Subsequently, the cells enter the subarachnoid space through an extension of the olfactory filaments as they traverse the sieve plate. The specific histological features of these nasal structures facilitate the direct entry of cells into the brain (Galeano et al., 2018; Li et al., 2022). The IN delivery is non-invasive, has relatively simple administration procedures, and will not cause additional harm to patients. Consequently, patients can tolerate high-frequency administration through IN route (Chau et al., 2018). However, the unique and complex structure of the nasal cavity, along with its blood circulation, active enzymes, and defense mechanisms, creates significant challenges for delivering drugs to the brain through IN route (Miyake and Bleier, 2015). As a result, further preclinical studies are essential to provide sufficient safety and efficacy data. This research is crucial to confirm that stem cells can effectively reach the targeted areas of the brain when administered intranasally, paving the way for future clinical trials.

#### 5.1.2 Modeling of BBB *in vitro*

In addition to their use as a therapeutic modality, human pluripotent stem cells (hPSCs) can be derived to mimic the characteristics of the human BBB for application in brain and organoid models. *In vitro* BBB modeling can reproduce both physiological and pathological states of BBB. Consequently, BBB modeling represents a valuable instrument for elucidating disease etiology and a foundation for evaluating prospective pharmaceutical agents. As a result, it has been utilized to anticipate the permeability of CNS drugs (Aday et al., 2016; Yan et al., 2021).

Human pluripotent stem cells can be differentiated into BMECs, pericytes and astrocytes, which can be jointly applied with *in vitro* BBB models. Induced pluripotent stem cells (iPSCs)-derived BMECs exhibit a mixture of endothelial and epithelial characteristics at the transcriptional level and lack the expressions of key endothelial adhesion molecules, such as vascular cell adhesion molecule-1 and intercellular adhesion molecule-2, which mediate the interaction between immune cells and BBB, as reported before (Nishihara et al., 2020; Lu et al., 2021). Consequently, researchers have devised the extended endothelial cell culture method (EECM) to iPSCs into BMECs with comparable morphology, barrier properties, and expression of endothelial adhesion molecules to those of primary human BMECs. BMEC-like cells reproduce numerous functional and molecular characteristics of BMECs *in vivo*, thus becoming a crucial instrument for elucidating the pathophysiological mechanisms behind dysfunction of BBB and identifying novel therapeutic targets for BBB injury (Lippmann et al., 2020; Matsuo et al., 2023).

The iPSC-derived BBB model is a straightforward, two-dimensional structural representation of neurovascular unit (NVU) cells that does not form intricate three-dimensional structures in specific brain regions. In contrast, the microfluidic BBB-on-chip model reproduces more complex structure and function of BBB *in vivo* by integrating multiple iPSC-derived cells so as to simulate the multicellular structure of BBB *in vitro*. The model can precisely control various factors, including three-dimensional vessel-like structures, cell-cell interactions, cell-ECM interactions, substrate stiffness, and mechanical shear (Yan et al., 2021). In comparison to conventional models, it offers advantages in terms of cell permeability imaging and real-time monitoring of trans-epithelial electrical resistance (TEER) values (Srinivasan et al., 2015). Therefore, the combination of iPSC technology with microfluidics can serve as an instrument with great promise for mechanobiological studies and drug screening.

#### 5.1.3 Interaction of stem cells with immune mechanisms

The interaction of stem cells with the immune system of the host is of equal importance in the process of recovery from a stroke. Stem cells have the capacity to modulate the function of local immune cells and alter the composition of the immune microenvironment, thereby promoting neuroprotection and regeneration (Verma et al., 2022). MSCs are also capable of influencing the immune microenvironment by modulating the function of macrophages, promoting tissue repair and regeneration (Manneken and Currie, 2023). This remodeling of the immune microenvironment has been shown to play a pivotal role in mitigating inflammation while promoting the formation of new blood vessels and the regeneration of nerve cells, thereby leading to significant improvements in functional recovery after stroke (Dekoninck and Blanpain, 2019). These observations underscore the potential of stem cell therapy in providing novel therapeutic approaches and strategies for BBB repair following stroke.

Stem cells have been shown to modulate the inflammatory response by secreting a variety of cytokines and exosome, which are the focus of immune mechanisms that promote the repair of BBB.

### 5.2 Mechanisms of stem cell repair of damaged BBB

Disruption of BBB is well accepted as a significant contributing factor to the damage and dysfunction of brain tissue in the aftermath of a stroke. Stem cells are capable of repairing the damaged BBB through a variety of mechanisms, primarily through anti-inflammatory effects, antioxidant effects, regulation of matrix metalloproteinases (MMPs), and modulation of intercellular communication.

#### 5.2.1 Anti-inflammatory effect

Ischemic foci in the aftermath of an acute ischemic stroke may release a considerable number of inflammatory cytokines, the accumulation of which represents a primary trigger for the destruction of BBB. Additionally, these factors can facilitate the recruitment of peripheral neutrophils, macrophages, and lymphocytes, which contribute to leukocyte infiltration and further promote inflammation, thereby leading to nerve damage and formation of a pro-inflammatory vicious cycle.

Numerous studies have identified mechanisms through which stem cells inhibit inflammatory responses when repairing damaged BBB tissue. The administration of human amniotic membrane-derived mesenchymal stem cells has been demonstrated to dose-dependently ameliorate neurological deficits caused by cerebral infarction. This is achieved by inhibiting the conversion of inflammatory cytokines from anti-inflammatory (M2-type) to pro-inflammatory (M1-type) in combination with by reducing the disruption of BBB and apoptosis in peri-infarcted areas (Yoshida et al., 2021). The inflammatory response occurring at BBB increases the absorption of VEGF by the endothelial cells in the brain. This process not only promotes the formation of new blood vessels, known as angiogenesis, but also raises the permeability of BBB itself (Croll et al., 2004). The transplantation of MSCs through gap junction-mediated cell-cell interactions has been demonstrated to inhibit VEGF uptake by brain endothelial cells *in vitro*, which in turn suppressed the inflammatory response in the brain (Kikuchi-Taura et al., 2021).

The nuclear transcription factor NF-κB acts as a regulatory factor with multiple transcriptional roles in a variety of cells in brain tissue. NF-κB is activated during cerebral ischemia reperfusion injury, and the resulting NF-κB activation exacerbates cerebral ischemic injury through multiple different mechanisms, including promotion of inflammation, induction of apoptosis, and mediation of free radical damage (Huang et al., 2010). In an animal model of spontaneous cerebral hemorrhage, bone marrow mesenchymal stem cells (BMSC)-secreted TSG-6 has been demonstrated to ameliorate injury of BBB by regulating activated astrocytes, which is achieved by suppressing NF-κB signaling pathway (Tang et al., 2021). An investigator in 2022 initially identified that MSC treatment through IA route after stroke exerted neuroprotective effects by modulating SIRT-1-mediated NF-κB pathways to attenuate inflammatory vesicle signaling and apoptosis (Sarmah et al., 2022). Aquaporin 4 (AQP4), the primary water channel protein in the mammalian brain, is found in astrocytes throughout the central nervous system and can control neurological water homeostasis. Besides, it shows an elevated expression in cerebral ischemia (Manley et al., 2000; Verkman et al., 2017). Furthermore, the investigator observed that administration of MSCs through IA route could modulate AQP4 by regulating expression of Protein kinase C-δ (PKC δ), thereby attenuating post-stroke edema (Datta et al., 2022). PKC δ exerts a significant regulatory effect on disruption of BBB following neuroinflammation, which is exacerbated by the downregulation of adhesion molecule expression and endothelial cell injury.

With regard to modulation of immune system and inflammatory response, the transplantation of MSCs following transient MCAO in ICR mice demonstrated a reduction in IgG leakage in brain parenchyma and a reversal of gap formation of ZO-1, occludin, and claudin-5 in tight junctions (Cheng et al., 2018). Upregulated IL-6 contributes to exacerbation of neuroinflammation in CNS-associated disorders. MSCs have been demonstrated to effectively suppress IL-6 secreted and released from immune cells. This is attributed to secretion of anti-inflammatory factors, including IL-10 and TGF-β, by activated microglia, astrocytes, and immune cells that have infiltrated the CNS. In addition, MSCs promote the production of regulatory T cells (Tregs), which exert an additional suppressive effect on secretion of IL-6 and contribute to reduction in neuroinflammation by playing an additional inhibitory role (Negi and Griffin, 2020; Kerkis et al., 2024). Neural stem cells (NSCs) may also mitigate ischemic injury by inhibiting a plethora of pro-inflammatory mediators. Above studies illustrate the pivotal role of disparate sources of stem cells in restoring the integrity and function of BBB following cerebral ischemia by modulating the extracellular microenvironment and attenuating neuroinflammation (Hamblin and Lee, 2021).

#### 5.2.2 Antioxidant effect

Increasing evidence indicates that oxidative stress-mediated damage to BBB is crucial in pathogenesis of various neurological disorders (Song et al., 2020). In this case, the antioxidant effects of MSCs following transplantation may become a promising strategy in repairing damage of BBB. In an *in vitro* cellular model of MCAO, CCR2-overexpressing MSCs demonstrated the capacity to restore BBB integrity by inhibiting degradation of tight junctions and excess ROS production. It can be attributed to partial mediation by redox-regulated peroxiredoxin-4 (PRDX4), a member of the PRDX antioxidant family, in restoring BBB integrity. Additionally, overexpression of CCR2 resulted in enhanced homing ability of MSCs (Huang et al., 2018). Moreover, Nrf2 emerges as a significant transcription factor governing antioxidant defense mechanisms, and the Nrf2 pathway is activated in astrocytes to safeguard themselves and their neighboring neurons from oxidative damage. After stimulation by oxidative stress, Nrf2 is released from Keap1 and transported to the nucleus. The RNAs and expressions of Nrf2 and heme oxygenase-1 (HO-1) were upregulated in astrocytes co-cultured with BMSCs. Additionally, MSC transplantation was observed to enhance the secretion of other antioxidant enzymes, such as HO-1, which were significantly ameliorated by the Cx43/Nrf2 signaling pathway, enhanced the antioxidant effects of astrocytes, and prevented them from apoptosis (Chen X. et al., 2020).

#### 5.2.3 Regulation of MMPs

An additional potential mechanism through which MSCs facilitate repair of BBB is modulating MMP activity (Klein and Bischoff, 2011). MMPs have been observed to degrade tight junctions in early stages following cerebral ischemia or cerebral hemorrhage. In particular, the upregulation of MMP9 has been demonstrated to promote neuroinflammation, which in turn leads to the destruction of BBB (Yang and Rosenberg, 2011; Kurzepa et al., 2014). The administration of transplanted MSCs following MCAO inhibited MMP9 upregulation, attenuated neuroinflammation, and weakened neutrophil infiltration through downregulating intercellular adhesion molecule-1 (ICAM-1) (Cheng et al., 2018). Furthermore, controlling MMP9 upregulation has been demonstrated to promote downregulation of AQP4 and PKC δ, which in turn attenuates vasogenic cerebral edema and thereby protects the nerves (Datta et al., 2022). In addition, MMP9 has been reported to degrade ZO-1. Some studies of hNSC transplantation in mice with cerebral ischemia found that downregulating MMP9 could improve ZO-1 degradation, which is conductive to maintaining the integrity of BBB (Hamblin and Lee, 2021). Notably, a study has proved an association between elevated MMP8 concentrations in serum and an increased risk of small vessel stroke (Jia et al., 2021). In a model of acute cerebral infarction, transplantation of BMSC resulted in a decrease in MMP8 level in cerebrospinal fluid, leading to a reduction in the size of ischemic lesions and an improvement in neurological outcome (Bai et al., 2024).

#### 5.2.4 Promotion of neovascularization and regulation of intercellular communication

After ischemic stroke, the Wnt signaling pathway is believed to play a crucial role in regulating neovascularization and repairing the damaged BBB (Tran et al., 2016). Research indicates that activating the Wnt/β-catenin signaling pathway encourages endothelial cell proliferation, migration, and lumen formation, which enhances neovascularization and improves blood flow to the brain (Fang et al., 2023; Wang J. et al., 2024). In the early stages following ischemic, the Wnt signaling pathway facilitates neovascularization by promoting the release of pro-angiogenic factors, such as VEGF, from endothelial cells while simultaneously inhibiting anti-angiogenic factors like endogenin. Additionally, this pathway can activate the AKT signaling pathway by reducing the expression of negative regulators, such as phosphatase and tensin homolog, which is vital for maintaining BBB integrity (Cheng et al., 2023). In summary, the neovascularization triggered by the activation of the Wnt signaling pathway after a stroke not only aids in repairing the damaged BBB but also enhances the oxygen and nutrient supply to brain tissues, thereby fostering neurological recovery. Furthermore, transplanted MSCs secrete Wnt signaling-related ligands such as Wnt3a and Wnt7a, which activate the Wnt signaling pathway in endothelial cells. These Wnt signaling molecules promote the proliferation, differentiation, and migration of vascular endothelial cells, leading to vascular neovascularization and the repair of BBB (Lippmann et al., 2012).

*In vivo* and *in vitro* experiments in a rat model of MCAO demonstrated that treatment with MSCs promoted angiogenesis and vascular stabilization, while reducing BBB leakage. This was achieved through the facilitation of communication between BMECs and astrocytes via the VEGF/flk1 and angiopoietin 1 (Ang1)/Tie2 pathways (Zacharek et al., 2007; Yamaguchi et al., 2022). Furthermore, MSCs safeguard neural stem cells from neurotoxic agents by transferring functional mitochondria into damaged cells (Babenko et al., 2018; Paliwal et al., 2018).

## 6 Role of stem cell-derived exosomes on BBB after stroke

### 6.1 Stem cell-derived exosomes in treating stroke

**FIGURE 5:**
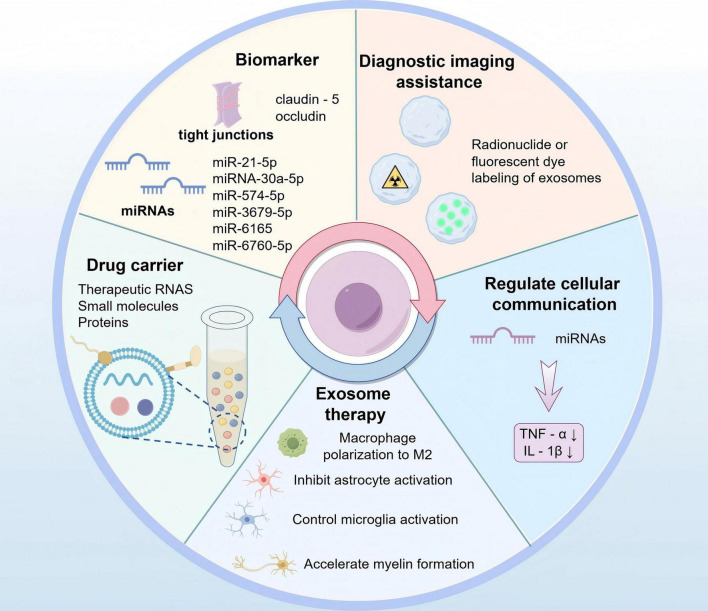
Exosomes in the diagnosis and treatment of BBB damage after stroke (by Figdraw).

There are increasing elucidation focusing on potential of using exosomes as mediators of intercellular communication in stroke therapy. Exosomes are nanoscale vesicles released by cells that contain different biologically active molecules, like proteins, mRNAs, miRNAs, and other components. The combinations of these molecules reflect the properties and physiological states of the cells from which they originate. Therefore, exosomes serve not only important mediators of intercellular communication but also perform varying functions within an organism (Mathivanan et al., 2011). The distinctive characteristics of exosomes have been significantly concerned due to their possibility of being applied in clinic treatment, particularly in the context of CNS diseases, where they may serve as biomarkers and carriers for diagnosis of agents (Liu et al., 2019; Figure 5).

#### 6.1.1 The diagnostic role of exosomes

Following a stroke, some exosomes derived from brain cells have been observed to cause detriment on the nervous system and they can traverse BBB and enter the peripheral blood and cerebrospinal fluid. Furthermore, those in bodily fluids may serve as biomarkers in diagnosing stroke and its prognosis, offering a non-invasive means of assessment (Li et al., 2018; Otero-Ortega et al., 2019). Numerous recent studies have indicated that microRNAs in exosomes may represent potential biomarkers for diagnosis of ischemic stroke (Jiang et al., 2022). What’s more, prior research has indicated that plasma-derived exosomes miRNAs, including miR-21-5p and miRNA-30a-5p, can be undertaken as biomarkers assisting in diagnosing ischemic stroke and differentiating its stage. This offers clinicians a novel reference for early determination of ischemic stroke and potential insights into the utility of biomarkers for thrombolytic therapy (Wang et al., 2018). Cerebrovascular disease represents a significant cause of stroke in Asian children and young adults. Expressions of several miRNAs, including miR-574-5p, miR-3679-5p, miR-6165, and miR-6760-5p, changed in exosomes targeting cerebrospinal fluid sources from moyamoya disease patients (Wang et al., 2020). In an earlier study, dysregulated miRNAs were also found to be closely associated with subarachnoid hemorrhage (Zheng et al., 2012). In conclusion, further attention is required to research focusing on ascertaining the use of exosomes as biomarkers to enhance their immediate diagnostic value for stroke.

#### 6.1.2 The therapeutic role of exosomes

Exosome therapy offers enhanced safety and convenience compared to traditional cell therapy, and can mitigate the risks associated with cell transplantation (Larson et al., 2024). However, exosomes derived from disparate sources exhibit disparate mechanisms and effects when they are applied for stroke treatment. Exosomes from BMSCs can protect nerves by modulating immune cell responses. Besides, exosomes contribute to polarization of microglia and macrophages toward M2-type while suppressing astrocyte activation. Additionally, exosomes can speed up myelination, thereby promoting neurovascular regeneration and protecting the nerves (Chai et al., 2024). Furthermore, adipose-derived mesenchymal stem cells (ADMSCs)-derived exosomes exhibit anti-inflammatory role and inhibitory effect on oxidative stress production in the presence of ischemia-reperfusion injury (Chen et al., 2016). After the brain-derived neurotrophic factor (BDNF) was loaded into NSC-derived exosomes, rats middle cerebral artery occlusion possessed BDNF-hNSC-Exos, which significantly enhances cell survival and facilitates endogenous NSCs differentiating into neurons, while concurrently controlling microglia activation (Zhang et al., 2023). Given the impact of cell type specificity on the biological properties of exosomes, it is imperative to identify the optimal source of exosomes for stroke treatment.

Currently, development of biomedical nanomaterials technology has transferred people’s attention to exploring various nanotechnology approaches for delivery of drugs to treat neurological complications. Actually, exosomes emerge as one such approach. Nanomedicines based on exosomes can encapsulate neuroprotective agents and anti-inflammatory compounds, which can penetrate BBB so as to deliver bioactive drugs to target cells with precision. Exosomes possess a unique cellular tropism and remarkable specificity in delivering drugs. In certain instances, exosomes achieve receptor-mediated tissue targeting through the modification of specific ligands on their surface (Zhang et al., 2022; Singh et al., 2024).

The clinical translation of exosome therapy is still confronted with numerous challenges, including the standardization of exosome preparation, dosage control, optimization of routes of administration, and the assessment of long-term safety. It is increasingly essential to investigate the specific mechanisms of exosomes in stroke treatment and develop more efficacious therapeutic strategies based on exosomes.

### 6.2 Role of stem cell-derived exosomes in BBB protection

Exosomes exert a pivotal role in physiological regulation of BBB through intricate communication among a multitude of cell types. Major mechanisms include repairing tight junctions, regulating miRNAs and reducing oxidative stress and apoptosis. There is mounting evidence to suggest that exosomes facilitate some crucial cellular communication processes by providing target cells with required functional proteins, metabolites, and nucleic acids, thereby influencing the BBB integrity after a stroke (Valadi et al., 2007; Men et al., 2019).

To illustrate, the neuronal release of miR-132-containing exosomes, which released by neurons and internalized and transported to vascular endothelial cells, could regulated through direct targeting of eukaryotic elongation factor 2 kinase by regulate vascular endothelial cadherin expression, enhance BBB integrity, which has been supported by previous studies (Xu et al., 2017). In addition, astrocyte-derived exosomes have also been demonstrated to contribute to maintaining function of BBB through releasing paracrine factors that regulate permeability and resistance of BMECs (Osaid et al., 2023). Furthermore, BMECs promote the formation of chemotactic gradients through exosome- and nanovesicle-mediated paracrine signaling, which induces the generation of nanotube structures and optimizes alignment of tight junctions among BMECs. This is critical for the robustness of BBB.

In the MCAO mice model, the researchers observed that exosomes exerted a protective effect by inhibiting apoptosis and autophagy in BMECs, as well as preserving the expressions of some tight junctions. Furthermore, exosomes enriched with MSC-derived miR-132-3p can reduce ROS production and BMEC apoptosis while maintaining the expressions of tight junctions, thereby conferring antioxidant and BBB protective benefits. This may be attributed to the inhibition of RASA1 expression by miR-132-3p in exosomes, which in turn activates the Ras/PI3K/Akt/eNOS signaling pathway (Pan et al., 2020). Exosomes have the potential to exert therapeutic effects by influencing BBB integrity following a stroke through modulation of multiple signaling pathways. A study investigating mice with ICH has demonstrated that hiPSC-NSC-Exos could reduce MCP-1 to release from astrocytes by over activating the PI3K/Akt/mTOR signaling pathway, thereby attenuating BBB disruption and ameliorating neurological deficits (Wang et al., 2025). Conversely, evidence suggests that stroke-induced damage to BBB is closely associated with exosomes containing the long non-coding RNA (lncRNA) H19, which are released by astrocytes. These exosomes can impair the function of BBB and even induce its dysfunction by downregulating expression of tight junctions through the lncRNA H19/miR-18a/VEGF signaling axis (Wang J. et al., 2022). In summary, these studies indicate that therapy using exosomes may enhance BBB integrity in stroke patients by regulating pertinent signaling pathways.

## 7 Neuroglia reprogramming

**FIGURE 6:**
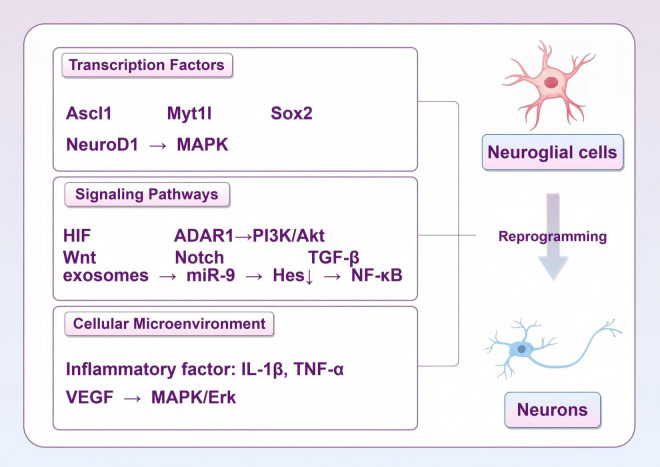
Strategies for reprogramming of glial cells into functional neurons (by Figdraw).

In nerve repair following stroke, stem cell therapy has been shown to promote nerve regeneration by supplying new neurons or support cells. Additionally, the reprogramming of glial cells has been observed to potentially generate endogenous support for nerve regeneration, with the combination of these approaches demonstrating the potential to enhance nerve repair (Tang et al., 2023; Williamson et al., 2023). Glial cells are capable of transforming into functional neurons under specific physiological and pathological conditions, a process that involves several key mechanisms (Figure 6). A comprehensive examination of these mechanisms offers novel insights and potential therapeutic strategies for neural repair after stroke.

### 7.1 Role of transcription factors

Dynamic regulation of transcription factors is one of the central mechanisms of cellular reprogramming after stroke. Using specific transcription factors, such as Ascl1, NeuroD1 and Myt1l, glial cells can be effectively converted into functional neurons (Liang et al., 2023). Overexpression of NeuroD1 effectively promotes the differentiation of NG2 glial cells into neurons, a process that is closely related to the activation of the MAPK signaling pathway (Wei et al., 2023). However, it has also been found that NeuroD1 does not effectively transform microglia into neurons, but rather induces apoptosis (Rao et al., 2021). SOX2, an important transcription factor, is able to regulate the properties of neural stem cells and promote the transformation of microglia and astrocytes into neurons (Hagey et al., 2022).

### 7.2 Regulation of signaling pathways

Signaling pathways play an equally important role in the reprogramming of glial cells into induced neurons. In the aftermath of ischemic injury, the hypoxia-inducible factor signaling pathway has been shown to promote metabolic reprogramming of glial cells, thereby enhancing their tolerance to ischemia and promoting neuronal survival (Madai et al., 2024). The PI3K/Akt signaling pathway has also been found to be closely associated with glial proliferation and transformation, and in particular, the activation of this pathway under the regulation of ADAR1 can promote the transformation of astrocytes into neurons (Cai et al., 2023). The response of astrocytes in inflammatory environments also affects their reprogramming ability through specific signaling pathways, suggesting that intercellular signaling is critical in this process (Zhou et al., 2022).

Signaling pathways such as Wnt, Notch, and TGF-β play key roles in the interactions between stem cells and glial cells (Azadian et al., 2023). The Wnt signaling pathway promotes the transition of glial cells to neurons by regulating their proliferation and differentiation (Freese et al., 2010). Notch signaling, on the other hand, plays an important role in maintaining the stem cell self-renewal and inhibits over-differentiation, which is essential for glial cell reprogramming (Zhou et al., 2010). In addition, the TGF-β signaling pathway plays an important role in regulating the inflammatory response and regeneration of glial cells, affecting their ability to transform into neurons. The interplay of these signaling mechanisms not only affects stem cell fate decisions, but also plays an important role in damage repair in the nervous system. Exosomes are enriched with miRNAs that modulate multiple signaling pathways, which in turn affect glial cell fate (Vahidinia et al., 2024). Exosomes affect glial cell proliferation and differentiation by regulating NF-κB and mTOR pathways (Szpakowski et al., 2023). Studies have shown that exosomes from mouse embryonic neural stem cells promote neural stem cell differentiation and inhibit their downstream target gene Hes1 by translocating miR-9, which in turn promotes neuron generation (Yuan et al., 2021). The modulation of these signaling pathways exerts a direct influence on cell survival and function, in addition to its involvement in the process of cell reprogramming. This provides a novel framework for the exploration of therapeutic targets for post-stroke neurological repair.

### 7.3 Influence of cellular microenvironment

The influence of the cellular microenvironment on the reprogramming of glial cells into induced neurons cannot be ignored. Inflammatory factors in the microenvironment such as IL-1β and TNF-α are able to promote the transformation of glial cells, leading to their transformation to neurons (Liang et al., 2023). Cytokines and signaling molecules in the inflammatory microenvironment also affect the reprogramming capacity of glial cells, and chronic low-grade inflammation can promote the supply of lactate to neurons but also impair its utilization efficiency (Wang Y. et al., 2022). In addition, interactions between astrocytes and microglia can regulate each other’s functions by secreting specific cytokines, thereby affecting the efficiency of cellular reprogramming (Tang et al., 2024). VEGF can promote astrocyte to neuron transformation through activation of the MAPK/Erk signaling pathway, suggesting the importance of the cellular microenvironment in the reprogramming process (Lei et al., 2023).

Comparison of stem cell therapy with reprogramming of glial cells into neurons provides new perspectives on neurological repair after stroke. Despite the challenges facing each of these two fields, their combination may provide more effective options for future therapeutic strategies.

## 8 Challenges and directions for future research

Recently, regenerative medicine and molecular biology have developed swiftly, which enables significant advancement for research on using stem cell and exosome therapies in stroke treatment. Such therapies in stroke treatment have been explored in a number of ways, including as therapeutic modalities, drug carriers and *in vitro* BBB modeling. It is imperative not to underestimate the significance of stem cells and exosomes in restoring BBB integrity following a stroke. The importance of stem cells and their exosomes in restoring BBB integrity after stroke cannot be overlooked.

As research into the use of stem cells and exosomes in neural repair continues, more evidences are emerging that these cells have beneficial effects on inflammation, apoptosis and neural regeneration. This suggests the significant potential of exosomes in repairing damaged BBB. In addition to reducing neuronal damage by regulating the microenvironment, exosomes can also promote recruitment and activation of endogenous stem cells, thereby assisting in the restoration of the function of damaged BBB.

While some articles have supported the role of stem cell exosomes in repairing BBB, it is imperative to acknowledge the discrepancies in perspectives across studies and the heterogeneity of research methodologies. It is recommended that future research concentrate on the systematic validation of the mechanism of action of stem cell exosomes, exploration of their effects in different stroke models, and the resolution of key issues such as the extraction and purification of stem cells and exosomes, cell sources, immune rejection, potential risk of tumor formation, and optimal routes of administration. Moreover, future research should concentrate on the creation of standardized therapeutic regimens. Meanwhile, clinical trials must be conducted with greater rigor to verify the safety and efficacy of these therapies through multicenter clinical trials. Only in this way, it will provide more compelling evidence for the clinical application of stem cell exosomes.

Advancement of these strategies to strengthen the specificity of targeting sites of neuroinflammation is imperative in the context of the complex architecture of the CNS, which requires the integration of advanced medical technologies and innovative engineering approaches, so that guarantee that stem cells or exosomes loaded with agents for treatments are efficiently and precisely delivered to the target brain region. To further the development of this field, it is essential to engage in multidisciplinary collaboration, integrating the resources of experts in diverse fields, including basic research, clinical application, and bioengineering. This approach enables a comprehensive examination of the biological properties and potential applications of stem cells and exosomes, spanning the full research continuum from fundamental biology to clinical translation.

In conclusion, studying stem cells and exosomes in repairing BBB after stroke is of significant scientific value and clinical significance. With further research and technological advancement, future studies will provide new insights and methodologies for early diagnosis of stroke, precise targeted therapy, and improved prognosis of stroke patients. Therefore, it is anticipated to achieve new developments in this field, which will lead to more effective treatment options for stroke patients.
